# Intra-cardiac thrombus resolution after anti-coagulation therapy with dabigatran in a patient with mid-ventricular obstructive hypertrophic cardiomyopathy: a case report

**DOI:** 10.1186/1752-1947-7-238

**Published:** 2013-10-08

**Authors:** Bunji Kaku

**Affiliations:** 1 Division of Cardiovascular Medicine, Toyama Red Cross Hospital, 2-1-58 Ushijima-honmachi, Toyama, Japan

**Keywords:** Dabigatran, Intra-cardiac thrombus, Mid-ventricular obstructive hypertrophic cardiomyopathy

## Abstract

**Introduction:**

Although dabigatran, a novel oral anti-coagulant, has been approved for the prevention of thromboembolism in patients with non-valvular atrial fibrillation, the efficacy of dabigatran for the resolution of established intra-cardiac thrombi has not been validated. Herein is describe a case in which dabigatran was effective for thrombus resolution in a patient with a left ventricular aneurysm.

**Case presentation:**

A 59-year-old Japanese man with a mid-ventricular obstructive hypertrophic cardiomyopathy-associated apical aneurysm presented with a left ventricular apical thrombus (15.0mm×17.0mm). Anti-coagulation therapy with dabigatran (150mg b.i.d. with meals) was initiated. Following dabigatran administration, weekly echocardiographic examinations demonstrated gradual decreases in thrombus size. After three weeks, no thrombus was detected and no systemic thromboembolic events had occurred.

**Conclusions:**

The left ventricular apical thrombus resolved after dabigatran administration. Hence, dabigatran may represent an alternative to warfarin as a therapeutic option in patients with previously detected intra-cardiac thrombus.

## Introduction

In general, heparin and warfarin are used for the treatment of intra-cardiac thrombi. Dabigatran is a novel oral anti-coagulant that has recently been approved for use in the prevention of thromboembolism in patients with non-valvular atrial fibrillation. However, because of limited dabigatran use in such situations, there are relatively few reports in the literature regarding the efficacy of dabigatran for the resolution of established intra-cardiac thrombi. Herein described is the effectiveness of dabigatran for thrombus resolution in a patient with a left ventricular aneurysm.

## Case presentation

**Figure 1:**
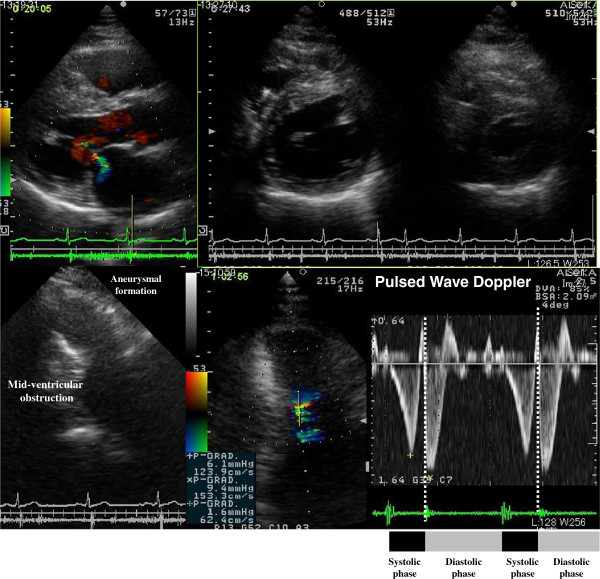
Echocardiographic images demonstrate asymmetrical septal hypertrophy and mid-ventricular obstruction associated with paradoxical diastolic flow.

**Figure 2:**
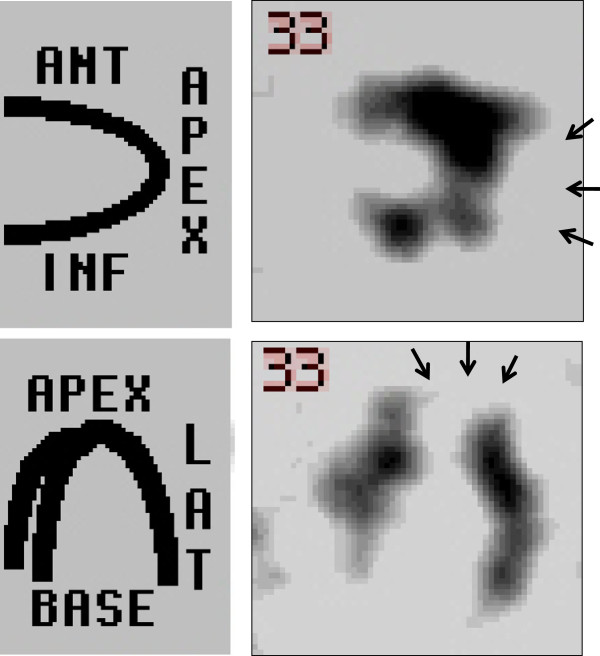
Technetium-99m tetrofosmin single-photon emission computed tomography scan demonstrates an area of severe hypoperfusion at the left ventricular apex (arrows).

**Figure 3:**
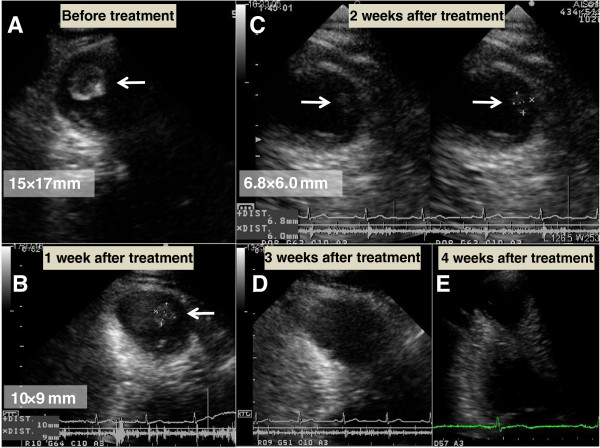
**Thrombic progression in apical aneurysm (thrombus indicated by arrows). (A)** Prior to anti-coagulation therapy, thrombus size was 15.0mm×17.0mm. **(B)** One week after initial dabigatran administration (150mg b.i.d.), thrombus size was 10.0mm×9.0mm. **(C)** Two weeks after initial dabigatran administration (150mg b.i.d.), thrombus size was 6.8mm×6.0mm. **(D)** Three weeks after initial dabigatran administration (150mg b.i.d.), thrombus was undetectable. **(E)** Four weeks after initial dabigatran administration (150mg b.i.d.), thrombus was still undetectable.

**Figure 4:**
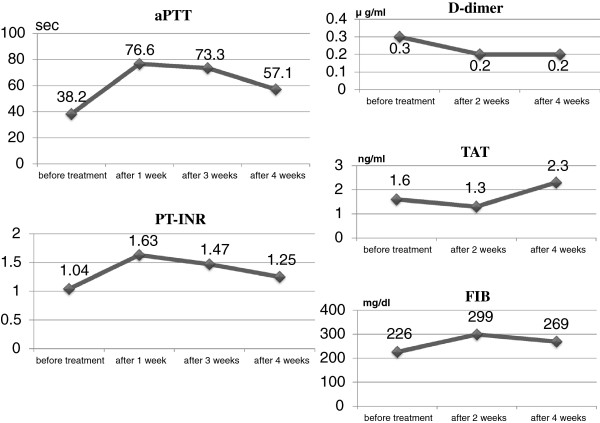
**Serial changes in coagulation markers before and after anti-coagulation therapy with dabigatran (150mg b.i.d.).** aPTT, activated partial thromboplastin time; PT-INR, prothrombin time internationalized ratio; TAT, thrombin-anti-thrombin III complex; FIB, fibrinogen.

A 59-year-old Japanese man presented for treatment of an intra-cardiac thrombus. At 52 years of age, he was diagnosed with mid-ventricular obstructive hypertrophic cardiomyopathy (HCM) associated with paroxysmal atrial fibrillation. Electrocardiography revealed poor R-wave progression at the V4 and V5 leads, and echocardiographic images demonstrated asymmetrical septal hypertrophy and mid-ventricular obstruction associated with paradoxical diastolic flow (Figure 1). Although the global left ventricular ejection fraction was not markedly impaired, aneurysm formation was observed at the left ventricular apex (Figure 1), and technetium (99mTc) tetrofosmin single-photon emission computed tomography demonstrated severe hypoperfusion in this area (Figure 2). Coronary angiography was concurrently performed, but revealed no atherosclerotic lesions in the coronary arteries. At 55 years of age, the patient developed sustained ventricular tachycardia associated with hemodynamic collapse, for which an implantable cardioverter-defibrillator (ICD) was inserted. Although the administration of bisoprolol (5mg/day) and amiodarone (200mg/day) were continued for the suppression of ventricular tachycardia after ICD implantation, anti-coagulation therapy was not initiated because no thrombus was detected on two echocardiographic examinations of the patient at 52 and 55 years of age. During the most recent echocardiographic examination, however, an asymptomatic thrombus (15.0mm×17.0mm) was detected in the apical aneurysm (Figure 3). Therefore, hospital admission was recommended for anti-coagulation therapy with continuous heparin infusion and warfarin administration. However, because the patient obstinately declined hospital admission and requested the continuation of medical treatment at an ambulatory clinic, dabigatran administration (150mg b.i.d.) was initiated. Coagulation markers were measured before the commencement of anti-coagulation therapy, and all were within the normal range (Figure 4). Following dabigatran administration, an approximately two-fold increase in activated partial thromboplastin time (measured four hours after dabigatran administration with meals) was observed and the prothrombin time internationalized ratio (PT-INR) was increased (Figure 4). However, the levels of D-dimer, thrombin-anti-thrombin III complex (TAT) and fibrinogen were not significantly altered (Figure 4). Moreover, weekly echocardiographic examinations demonstrated a gradual decrease in thrombus size (Figure 3). Echocardiographic examinations performed one week after initial dabigatran administration showed that thrombus size had decreased to 10.0mm×9.0mm. Thrombus size had further decreased to 6.8mm×6.0mm after two weeks, and no thrombus was detected after three and four weeks. During the four-week treatment period, no systemic thromboembolic events occurred. Thus, the thrombus appears to have been resolved after dabigatran administration. However, dabigatran administration was continued even after thrombus resolution.

## Discussion

Mid-ventricular obstructive hypertrophic cardiomyopathy is a rare form of HCM, accounting for approximately 5% of all HCM cases [1]. However, it is sometimes associated with apical aneurysms without significant atherosclerotic coronary artery stenosis [2]. The annual incidence of adverse cardiovascular events, including sudden death, appropriate ICD discharges, thromboembolic stroke and progressive heart failure, has been reported to be 10.5% in patients with HCM-associated apical aneurysms [2]. Maron *et al*. reported the clinical courses of 28 patients with HCM-associated apical aneurysms over a mean follow-up period of 4.1±3.7 years, in which embolic stroke or left ventricular apical thrombus was detected in four patients [2]. On the basis of these results, they recommended prophylactic anti-coagulation therapy for the prevention of embolic stroke in patients with apical aneurysms. In the present case, no apical thrombus was detected on echocardiographic examinations of the patient at 52 and 55 years of age, and anti-coagulation therapy was not initiated. However, despite the patient’s being asymptomatic, left ventricular apical thrombus was detected on his echocardiographic examination performed at 59 years of age.

Dabigatran is a reversible direct thrombin inhibitor which has been approved for the prevention of thromboembolism in patients with non-valvular atrial fibrillation [3] and in those with thromboembolism after hip and knee replacement surgeries [4]. Thrombin is a key factor in the coagulant process because it converts fibrinogen into fibrin and activates platelets and factors V, VII, VIII, IX and XIII. Following oral administration, dabigatran etexilate is rapidly hydrolyzed into dabigatran and subsequently absorbed from the gastrointestinal tract. Dabigatran inactivates free thrombin and fibrin-bound thrombin in a concentration-dependent manner [5]. The efficacy and safety of dabigatran for the prevention of stroke or systemic embolism in patients with non-valvular atrial fibrillation was shown in the Randomized Evaluation of Long-term Anticoagulation Therapy trial [3]. In that trial, dabigatran administration at 110mg b.i.d. was associated with similar rates of stroke and systemic embolism, but with a lower rate of major bleeding than that associated with warfarin. At 150mg b.i.d., dabigatran was associated with lower rates of stroke and systemic embolism, but similar rates of major bleeding, compared with those of warfarin. Furthermore, the incidence of intra-cranial bleeding was significantly lower during the anti-coagulation therapy with both dabigatran doses compared with those of warfarin. In patients with non-valvular atrial fibrillation, in whom atrial thrombus was identified on transesophageal echocardiography, the atrial thrombus was resolved by four weeks of warfarin administration in approximately 80% to 90% of cases [6,7].

In contrast, the efficacy of the oral anti-coagulant dabigatran in the resolution of previously detected thrombus has not been extensively reported. Vidal *et al*. [8] reported the first documented case of thrombus resolution following dabigatran administration. In their report, after dabigatran administration (150mg b.i.d.) for eight weeks, a large left atrial appendage thrombus was resolved with no concomitant thromboembolic events. In the present case, on the basis of weekly echocardiographic examinations, the size of the apical aneurysm thrombus gradually decreased. After dabigatran administration for three weeks, the apical aneurysm thrombus disappeared without any thromboembolic events. The absence of clinical events made migration of the thrombus an unlikely explanation for its disappearance. Because plasma levels of fibrinogen and TAT did not increase, systemic coagulation activities did not increase, despite the presence of a left ventricular thrombus. Before and after anti-coagulation therapy with dabigatran, the plasma D-dimer level, which is a fibrinolysis marker, did not increase. After dabigatran administration, activated partial prothrombin time was moderately prolonged and PT-INR was slightly extended. These findings suggest that, in situations in which dabigatran suppresses thrombin activity without the activation of fibrinolysis, endogenous fibrinolysis and the prevention of new thrombus formation by dabigatran administration may be the primary mechanism of thrombus resolution. Some patients showed resistance to anti-coagulation therapy with warfarin [8], which also inhibits vitamin K-dependent γ-carboxylation of proteins C and S. Therefore, warfarin also has a potential thrombogenic effect by inhibiting the activities of these anti-coagulant proteins. It was considered that the initiation of warfarin therapy without the addition of heparin at the ambulatory clinic may have triggered this warfarin-induced proteins C and S paradox. Therefore, dabigatran may present some advantages over warfarin. The patient had the habit of ardently eating natto, a traditional Japanese fermented soybean dish. Natto contains a large quantity of vitamin K; thus, consumption of even a small quantity of natto can strongly reverse the effects of warfarin. Upon initiation of anti-coagulation therapy with warfarin, patients must completely avoid consuming vitamin K-rich foods such as natto. The patient did not adhere to this suggestion, although he was informed that the success of the treatment was dependent on his not consuming natto. In contrast, dabigatran efficacy is not limited by this type of food. If dabigatran efficacy in the resolution of intra-cardiac thrombus can be confirmed in other cases, dabigatran may represent an alternative therapeutic option to warfarin in patients with previously detected intra-cardiac thrombus.

## Conclusions

Because thrombus resolution was successful in the present case, dabigatran may have the potential to be used as an alternative to warfarin in patients with established intra-cardiac thrombus. However, additional cases are needed to determine if dabigatran is effective for the resolution of intra-cardiac thrombus.

## Consent

Written informed consent was obtained from the patient for publication of this case report and accompanying images. A copy of the written consent is available for review by the Editor-in-Chief of this journal.

## Abbreviations

PT-INR: Prothrombin time internationalized ratio; TAT: Thrombin-anti-thrombin III complex.

## Competing interests

The author declares that he has no competing interests.

## Author’s contribution

BK analyzed and interpreted the patient data and wrote the manuscript.
